# Glucagon-like Peptide-1 Receptor Agonist Therapy and Risk of Pulmonary and Systemic Infections in Diabetic Gastroparesis: A Propensity-Matched Cohort Study

**DOI:** 10.3390/arm94020020

**Published:** 2026-03-24

**Authors:** Muhammad Ali Ibrahim Kazi, Hasan Kamal, Syed Musa Mufarrih, Imran Qureshi, Sanmeet Singh, Adrien Mazer

**Affiliations:** 1 Luminis Health Anne Arundel Medical Center, Annapolis, MD 21401, USA; hkamal@luminishealth.org (H.K.); ssingh4@luminishealth.org (S.S.); amazer1@luminishealth.org (A.M.); 2 Medical College, Aga Khan University, Karachi 74800, Pakistan; syed.mufarrih@scholar.aku.edu; 3 Department of Internal Medicine, Rutgers New Jersey Medical School, Newark, NJ 07103, USA

**Keywords:** GLP-1 receptor agonists, diabetic gastroparesis, pneumonia, sepsis, TriNetX, propensity score matching, infectious complications

## Abstract

**Highlights:**

**What are the main findings?**

- GLP-1 receptor agonist therapy was associated with reduced risk of pulmonary and systemic infections in patients with diabetic gastroparesis.
- Non-GLP-1 users had significantly higher rates of pneumonitis, pneumonia, sepsis, and bacteremia.

**What are the implications of the main findings?**

- GLP-1 receptor agonists may be safer than previously assumed in patients with gastroparesis.
- Their anti-inflammatory and metabolic benefits may outweigh concerns about delayed gastric emptying.

**Abstract:**

Introduction: Diabetic gastroparesis increases the risk of aspiration, pneumonia, and sepsis, yet the impact of glucagon-like peptide-1 receptor agonists (GLP-1 RAs) on these outcomes is uncertain because of their gastric-emptying effects. Methods: We performed a retrospective cohort study using the TriNetX Global Research Network. Adults (≥18 years) with diabetes mellitus and gastroparesis were identified and divided into two cohorts based on GLP-1 RA exposure. Propensity score matching (1:1) balanced demographics, comorbidities, and antidiabetic medications, yielding 23,371 patients per cohort. Outcomes, assessed from 180 days after index, included pneumonia, pneumonitis, mechanical ventilation, ventilator-associated pneumonia, sepsis, bacteremia, empyema, lung abscess, acute respiratory distress syndrome (ARDS), and need for enteral feeding. Risk ratios (RRs) and hazard ratios (HRs) with 95% confidence intervals (CIs) were estimated. Results: Compared with GLP-1 users, non-GLP-1 patients had higher incidences of pneumonitis (3.6% vs. 2.5%; HR 1.76, 95% CI 1.58–1.95), pneumonia (13.2% vs. 12.2%; HR 1.34, 95% CI 1.27–1.41), mechanical ventilation (4.4% vs. 3.3%; HR 1.63, 95% CI 1.49–1.79), sepsis (12.8% vs. 11.1%; HR 1.44, 95% CI 1.37–1.52), and bacteremia (5.2% vs. 4.4%; HR 1.46, 95% CI 1.35–1.59) (all *p* < 0.001). Empyema and ARDS were also numerically lower among GLP-1 users, while ventilator-associated pneumonia and lung abscess were rare and similar between groups. No patients required percutaneous endoscopic gastrostomy or nasal enteral feeding. Conclusions: In patients with diabetes and gastroparesis, GLP-1 RA therapy was associated with significantly fewer pulmonary and systemic infectious complications. These data suggest that the systemic benefits of GLP-1 RAs may outweigh concerns regarding delayed gastric emptying in this high-risk population.

## 1. Introduction

Diabetic gastroparesis is a chronic complication of diabetes characterized by delayed gastric emptying in the absence of mechanical obstruction, resulting in nausea, vomiting, early satiety, bloating, and glycemic fluctuations [1,2,3]. It represents a clinical intersection between gastrointestinal dysmotility and metabolic dysfunction, commonly arising from autonomic neuropathy and microvascular injury [2,3,4]. The condition significantly impairs quality of life, leads to frequent emergency visits, and complicates glycemic management, as erratic nutrient absorption disrupts postprandial glucose control [5,6,7].

Delayed gastric emptying increases gastric residual volume and predisposes to regurgitation and aspiration [8,9]. Consequently, affected patients are at higher risk for aspiration pneumonia, pneumonitis, and prolonged ventilatory support [8,10]. Additionally, diabetes-related immune dysfunction—marked by impaired neutrophil activity, microvascular compromise, and chronic inflammation—further amplifies susceptibility to bacterial infections and sepsis [11,12]. Glucagon-like peptide-1 receptor agonists (GLP-1 RAs) improve glycemic control, inducing weight loss, and reducing major cardiovascular and renal events [13,14]. However, their pharmacologic effect on gastric motility has raised concern regarding their use in patients with or at risk for gastroparesis [15,16,17]. GLP-1 RAs can exacerbate postprandial fullness and nausea in susceptible individuals [15,16]. Interestingly, GLP-1 RAs also possess anti-inflammatory and cytoprotective properties that may counterbalance their gastrointestinal motility effects. Experimental studies have demonstrated that GLP-1 activation reduces systemic inflammation and mitigates oxidative stress—all of which could theoretically lower susceptibility to infectious complications [18,19]. In addition, improved metabolic control and weight reduction may indirectly reduce infection-related morbidity and hospitalizations in patients with long-standing diabetes [13,14,15].

Given the rising use of GLP-1 receptor agonists and the increasing prevalence of diabetic gastroparesis, understanding the broader safety and outcome profile of these agents is of growing clinical importance. While prior research has focused predominantly on cardiovascular and metabolic endpoints, their effects on respiratory and infectious outcomes remain largely unexplored [9,17,18,19].

### Objective

To examine the association between glucagon-like peptide-1 receptor agonist (GLP-1 RA) exposure and the subsequent risk of selected pulmonary and systemic infectious outcomes among adults with diabetes and gastroparesis, using a retrospective, propensity score-matched cohort from a large real-world electronic health record network.

## 2. Methods

### 2.1. Data Source and Study Design

We conducted a retrospective cohort study using the TriNetX Global Research Network (TriNetX LLC, Cambridge, MA, USA), a federated, real-world data platform providing access to de-identified electronic health records (EHRs) from over 100 million patients across 102 healthcare organizations (HCOs) [20,21,22]. Data within TriNetX include demographics, diagnoses (International Classification of Diseases, Ninth and Tenth Revision, Clinical Modification [ICD-9/10-CM]), procedures (Current Procedural Terminology [CPT]), medications (Anatomical Therapeutic Chemical [ATC] classification), and laboratory results (Logical Observation Identifiers Names and Codes [LOINC]).

The study adhered to the Declaration of Helsinki and was deemed exempt from institutional review board (IRB) review due to the use of de-identified data under 45 CFR 46.104(d)(4).

### 2.2. Study Population and Cohort Definitions

Adults aged ≥18 years with documented diagnoses of diabetes mellitus (DM) (ICD-10: E08–E13) and gastroparesis (ICD-10: K31.84) were included. Patients were divided into two cohorts based on exposure to glucagon-like peptide-1 receptor agonists (GLP-1 RAs), defined by Anatomical Therapeutic Chemical (ATC) classification A10BJ:

- **Cohort 1 (DM + Gastroparesis − GLP-1):** Patients with Diabetes Mellitus and gastroparesis who had no record of GLP-1 RA exposure.
- **Cohort 2 (DM + Gastroparesis + GLP-1):** Patients with Diabetes Mellitus and gastroparesis with recorded GLP-1 RA use.

The **index date** was defined as the first date meeting all cohort-specific inclusion criteria. Outcomes were assessed beginning 180 days after the index date to avoid immortal time bias. Patients with index events older than 20 years were excluded. Patients included in the study had GLP-1 therapy initiated prior to the diagnosis of gastroparesis in all patients.

Inclusion criteria were:

1. Age ≥ 18 years at index date;
2. Confirmed diagnosis of both DM and gastroparesis;
3. Availability of complete demographic and clinical data;
4. Minimum 6-month follow-up post-index.

### 2.3. Outcomes

Primary and secondary outcomes were identified using validated ICD-10 and CPT codes representing pulmonary and systemic infectious complications, including:

- Pneumonia (J13–J18; 483);
- Pneumonitis (J69);
- Ventilator-associated pneumonia (VAP) (J95.851);
- Mechanical ventilation use (CPT 1015098);
- Sepsis (A40–A41);
- Empyema (J86);
- Lung abscess (J85);
- Acute respiratory distress syndrome (ARDS) (J80);
- Bacteremia (R78.81);
- Percutaneous endoscopic gastrostomy (PEG) or enteral feeding (SNOMED 229912004, 183028005).

Each outcome was evaluated separately, and patients were analyzed from the index date until first occurrence of outcome, death, loss to follow-up, or end of the observation window, whichever occurred first.

### 2.4. Propensity Score Matching and Covariates

To mitigate confounding, cohorts were propensity-score matched (PSM) at a 1:1 ratio using a greedy nearest-neighbor algorithm without replacement and a caliper of 0.1 standard deviations of the logit of the propensity score. Covariates included demographics (age, sex, race, ethnicity), comorbidities (hypertension, obesity, chronic kidney disease, coronary artery disease, chronic obstructive pulmonary disease, liver disease, neoplasms), and concurrent antidiabetic medications (insulin, metformin, sulfonylureas, DPP-4 inhibitors, SGLT2 inhibitors, and thiazolidinediones).

Standardized mean differences (SMDs) were used to assess balance, with SMD < 0.1 considered acceptable. After matching, both cohorts included 23,371 patients, achieving near-perfect covariate balance across all baseline characteristics.

### 2.5. Statistical Analysis

All analyses were performed within the TriNetX Analytics environment using validated statistical engines built in R (v4.0.2; *survival* v3.2-3, *Hmisc* v4.1-1), Python (v3.7; *lifelines* v0.22.4, *statsmodels* v0.13.2, *pandas* v1.3.5), and Java (v11.0.16).

Risk analyses estimated risk ratios (RRs), odds ratios (ORs), and risk differences (RDs) with 95% confidence intervals (CIs). Kaplan–Meier survival analyses and log-rank tests assessed differences in time-to-event distributions, while Cox proportional hazards models calculated hazard ratios (HRs) with robust sandwich variance estimators clustered by matched pair.

The proportional hazards assumption was tested using Schoenfeld residuals and inspection of log(–log) survival plots. All statistical tests were two-tailed, with significance defined as *p* < 0.001. *This conservative threshold was selected to mitigate the risk of type I error in the context of a large sample size and multiple outcome assessments. Formal adjustments for multiple comparisons were not applied; therefore, the stricter significance criterion was used as a pragmatic alternative to reduce false-positive findings.*

### 2.6. Ethical Considerations

The TriNetX platform ensures compliance with HIPAA and GDPR standards by using aggregated, de-identified data. As such, this study qualified for IRB exemption and did not require informed consent.

## 3. Results

### 3.1. Study Population and Cohort Characteristics

After applying inclusion criteria and 1:1 propensity score matching, a total of 46,742 patients were included in the final analysis, with 23,371 patients in each cohort:

- Cohort 1 (Diabetes Mellitus + Gastroparesis − GLP-1): patients with diabetes and gastroparesis without GLP-1 receptor agonist use.
- Cohort 2 (Diabetes Mellitus + Gastroparesis + GLP-1): patients with diabetes and gastroparesis with GLP-1 receptor agonist exposure.

Before matching, the unadjusted cohorts consisted of 116,272 and 30,669 patients, respectively. Propensity matching yielded excellent covariate balance across all demographic and clinical variables (standardized mean difference < 0.1).

**Figure 1:**
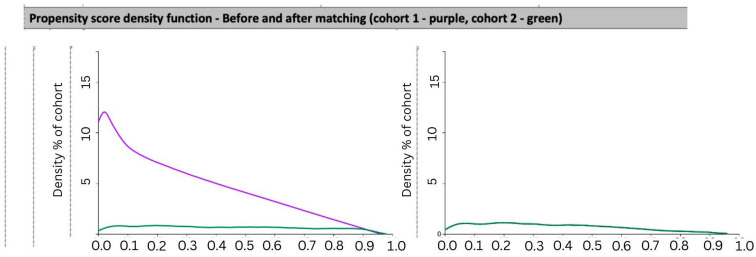
Propensity score density distribution for both cohorts before and after matching.

The mean age of patients after matching was 61 ± 14 years, and 69.3% were female. The racial and ethnic composition was comparable between groups, with 65% identifying as White and 20% as Black or African American. Major comorbidities such as hypertension, chronic kidney disease, and chronic liver disease were evenly distributed. Use of concurrent antihyperglycemics—including metformin, insulin, SGLT2 inhibitors, and DPP-4 inhibitors—was similarly balanced post-matching. The propensity score distribution before and after matching is shown in Figure 1.

### 3.2. Overview of Outcomes

Pulmonary and systemic infectious outcomes were evaluated beginning 180 days after the index date. Compared with GLP-1-treated patients, those without GLP-1 exposure demonstrated consistently higher rates of pneumonia, sepsis, pneumonitis, bacteremia, and ventilator-related complications.

### 3.3. Pneumonitis and Pneumonia

The incidence of pneumonitis was significantly higher in non-GLP-1 users (3.6%) compared with GLP-1 users (2.5%) (risk ratio [RR] 1.43, 95% CI 1.29–1.59; *p* < 0.001). Kaplan–Meier analysis demonstrated superior event-free survival among GLP-1-treated patients (84.1% vs. 75.2%; log-rank *p* < 0.001), corresponding to a hazard ratio (HR) of 1.76 [95% CI 1.58–1.95] for non-users.

Similarly, pneumonia occurred in 13.2% of non-GLP-1 users versus 12.2% of GLP-1 users (RR 1.08, 95% CI 1.03–1.13; *p* = 0.002). Median survival time was prolonged in GLP-1 users (not reached vs. 5683 days), with survival probabilities of 53.4% versus 41.2%, respectively. The corresponding hazard ratio was 1.34 (95% CI 1.27–1.41; *p* < 0.001), indicating a sustained reduction in pneumonia-related events with GLP-1 therapy.

### 3.4. Ventilator-Associated Outcomes

The risk of mechanical ventilation or ventilator management was higher in non-GLP-1 users (4.4%) compared with those treated with GLP-1 agents (3.3%) (RR 1.33, 95% CI 1.22–1.46; *p* < 0.001). The Kaplan–Meier survival probability at end of follow-up was 84.6% for GLP-1 users versus 80.7% for non-users, yielding an HR of 1.63 (95% CI 1.49–1.79). Ventilator-associated pneumonia (VAP) was rare in both groups (0.2%), with no statistically significant difference (*p* = 0.75).

### 3.5. Sepsis and Bacteremia

Systemic infectious complications showed the largest divergence between cohorts. Sepsis occurred in 12.8% of non-GLP-1 users and 11.1% of GLP-1 users (RR 1.16, 95% CI 1.10–1.22; *p* < 0.001). Median survival for sepsis-free status was markedly longer in GLP-1-treated patients (not reached vs. 6258 days), and the HR for sepsis in non-users was 1.44 (95% CI 1.37–1.52; *p* < 0.001).

Similarly, bacteremia was more common among non-users (5.2% vs. 4.4%), with a risk ratio of 1.19 (95% CI 1.10–1.29; *p* < 0.001) and an HR of 1.46 (95% CI 1.35–1.59). Event-free survival at the end of follow-up was 75.0% for non-GLP-1 users versus 81.5% for GLP-1 users (*p* < 0.001 by log-rank).

### 3.6. Pulmonary Complications

Less-frequent but clinically significant respiratory complications were also examined. The incidence of empyema was 0.4% in non-users and 0.3% in GLP-1 users (RR 1.49, 95% CI 1.10–2.03; *p* = 0.011), whereas lung abscess and ARDS did not differ significantly between groups (*p* = 0.64 and *p* = 0.16, respectively). The HR for ARDS was 1.41 (95% CI 1.15–1.74; *p* = 0.001), suggesting a modest trend toward reduction with GLP-1 therapy.

### 3.7. PEG Tube and Nutritional Support

No patients in either group required percutaneous endoscopic gastrostomy (PEG) or nasal enteral tube feeding during the study period, consistent with the low frequency of severe gastroparesis necessitating invasive nutritional intervention.

### 3.8. Summary of Comparative Outcomes

Kaplan–Meier analysis demonstrated a clear separation between the two study cohorts over the follow-up period. Patients in the control group experienced a consistently lower event-free survival probability compared with those in the GLP cohort. By the end of the observation window, the estimated survival probability was 45.9% in the normal cohort versus 61.3% in the GLP cohort. The median time to event was reached only in the normal group (6258 days), whereas it was not reached in the comparison cohort, indicating more prolonged event-free survival. This difference between curves was statistically significant (*p* < 0.0001). In time-to-event modeling, the normal cohort had a 44% higher hazard of the outcome compared with the GLP cohort (hazard ratio 1.44, 95% CI 1.37–1.52, *p* < 0.0001), confirming a significantly increased risk over time.

**Figure 2:**
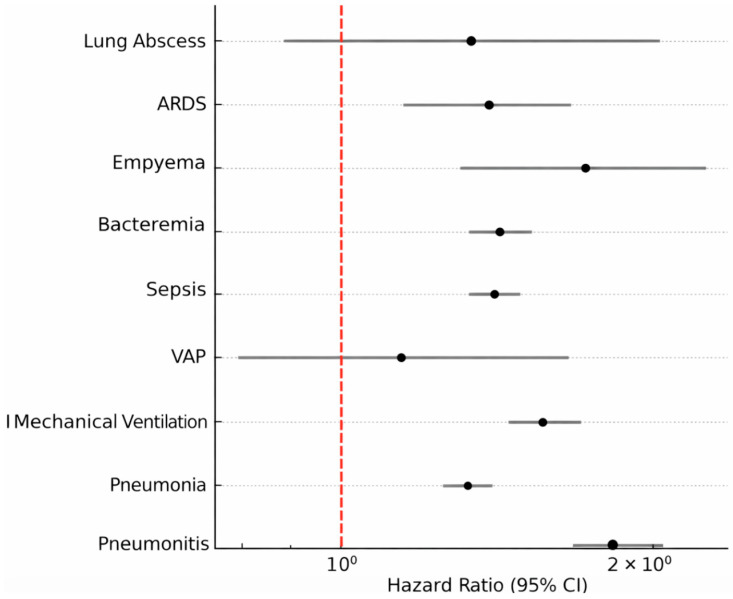
Forest plot illustrating hazard ratios with 95% confidence intervals for pulmonary and infectious outcomes in patients with diabetes and gastroparesis receiving versus not receiving GLP-1 therapy. The dashed vertical line indicates the null value” (hazard ratio = 1).

A comprehensive summary of risk and survival analyses is presented in Table 1. Detailed comparative outcomes, including risk ratios, hazard ratios, and absolute risk differences, are summarized in Table 2. Additional analyses and supporting data are provided in the Supplementary Materials (Tables S1 and S2). Across nearly all endpoints, GLP-1 receptor agonist therapy was associated with a 10–40% relative reduction in pulmonary and infectious complications compared with non-use. Figure 2 illustrates the corresponding forest plot of hazard ratios, demonstrating consistent protective associations favoring GLP-1 therapy across the evaluated outcomes. The results of severity-restricted sensitivity analyses were directionally consistent with the primary findings, though effect sizes were attenuated, suggesting that residual confounding related to disease severity cannot be excluded.

## 4. Discussion

In this large, real-world, propensity-matched analysis of patients with diabetes and gastroparesis, GLP-1 receptor agonist therapy was associated with significantly lower risks of pulmonary and systemic infectious complications, including pneumonitis, sepsis, and bacteremia. [23,24,25,26] These findings suggest that, despite theoretical concerns regarding delayed gastric emptying, GLP-1 receptor agonist use may confer a net protective effect in this high-risk population. The results also highlight an important but underexplored clinical interface between gastrointestinal dysmotility, infection risk, and metabolic therapy [23,27].

### 4.1. Pathophysiological Interpretation

The mechanisms underlying the observed protective association are likely multifactorial. GLP-1 receptor activation has well-documented metabolic benefits, including improved glycemic stability, weight reduction, and decreased postprandial glucose excursions, and also exerts direct anti-inflammatory and endothelial-protective effects [27,28,29,30].

While GLP-1 receptor agonists can transiently slow gastric motility and aggravate upper-gastrointestinal symptoms in some patients, the degree of delay is often modest and may attenuate with continued therapy [15,16,17]. Furthermore, patients in our study receiving GLP-1 therapy experienced fewer pulmonary infections and episodes of sepsis, aligning with emerging data that incretin-based regimens can reduce severe respiratory events and sepsis-related outcomes in high-risk diabetic populations [23,24,25,26,31].

### 4.2. Pulmonary and Infectious Outcomes

Among the outcomes evaluated, pneumonitis and sepsis demonstrated the strongest inverse associations with GLP-1 therapy. This is particularly relevant given that gastroparesis predisposes to micro-aspiration and respiratory compromise. Our study shows associations in the incidence of pneumonitis and pneumonia among GLP-1 users in our analysis that are concordant with large cohort and network-based studies showing that GLP-1 receptor agonists are associated with lower risks of pneumonia, severe sepsis, invasive mechanical ventilation, and respiratory failure [23,24,25,26,32,33]. The relationship of pneumonitis incidence suggests that these agents may potentially mitigate downstream pulmonary inflammation. Several secondary outcomes, including ventilator-associated pneumonia and lung abscess, demonstrated neutral associations, underscoring the need for cautious interpretation and further validation. Although these outcomes are relatively uncommon, they reflect critical-illness trajectories often precipitated by severe infection or aspiration events. The attenuation of such complications suggests that GLP-1 therapy may influence not only infection incidence but also infection severity and recovery potential [23,24,25,26,32,33].

### 4.3. Clinical Implications

Although these agents have historically been avoided in this subgroup because of concerns about worsening gastric motility, our data—together with accumulating real-world and perioperative evidence—suggest that the overall balance of risk and benefit may favor continued or cautious initiation in appropriately selected patients [23,24,27,32,33].

Second, these results underscore the broader systemic benefits of GLP-1 receptor agonists beyond glucose control. Their anti-inflammatory, endothelial, and immune-modulating effects may be clinically meaningful in reducing infection-related hospitalizations and complications, complementing the established cardiovascular and renal advantages reported in prior outcome trials [27,28,29,30,31].

### 4.4. Comparison with Prior Studies

While previous investigations have primarily focused on cardiovascular and renal outcomes of GLP-1 receptor agonists, our analysis extends these observations into infectious domains. Large outcome trials and mechanistic studies have shown that GLP-1 receptor agonists reduce markers of systemic inflammation [27,28,29,30,31]. Our findings are consistent with and complementary to recent real-world cohorts and meta-analyses suggesting that GLP-1-based regimens are associated with lower risks of pneumonia, sepsis, and respiratory complications, particularly among patients with chronic lung disease or multiple metabolic risk factors [23,24,25,32,33].

Notably, we did not observe an increase in severe gastroparesis-related complications among GLP-1 users. The absence of higher PEG-tube placement or escalation to mechanical feeding supports the notion that, in real-world settings, the risk of substantial motility worsening is relatively low compared with the potential systemic benefits [15,16,17]. This finding is clinically significant given long-standing hesitancy to prescribe GLP-1 receptor agonists in patients with any degree of gastric motility disorder and aligns with emerging peri-procedural data showing either neutral or modest effects on aspiration-related outcomes when GLP-1 agents are appropriately managed [32,33].

These findings derive from the TriNetX network and may not be generalizable to healthcare systems with different prescribing practices, population characteristics, or coding structures.

### 4.5. Strengths and Limitations

The strengths of this study include the use of a large, multicenter, real-world dataset encompassing diverse health systems and patient demographics, as well as rigorous propensity matching to minimize confounding. The sample size and event counts provided sufficient power to detect clinically meaningful differences across multiple infectious endpoints, and use of a federated network such as TriNetX allowed inclusion of patients from both academic and community settings [20,21,22,34].

However, several limitations should be acknowledged. First, as this was a retrospective observational study, causality cannot be inferred. Nonrandomized analyses of EHR-derived data are inherently susceptible to channeling bias, immortal time bias, and unmeasured confounding, even when advanced adjustment methods are employed [35]. Second, misclassification bias may have occurred. TriNetX relies on structured diagnosis and procedure codes rather than adjudicated clinical phenotypes, and does not fully capture treatment adherence, over-the-counter medication use, lifestyle factors, or social determinants of health—all of which can influence infection risk and outcomes [34,35].

Third, temporal relationships between GLP-1 initiation, gastroparesis onset or progression, and infectious events cannot be precisely established, as documentation frequency and follow-up intervals vary across health systems. Fourth, dose–response information and distinctions among individual GLP-1 agents were limited for many HCOs, precluding detailed comparative effectiveness analyses by molecule or dose. As a result, analyses were conducted at the class level, and potential heterogeneity in effects across individual agents or dosing strategies could not be evaluated. These findings should therefore be interpreted as class-level associations rather than evidence of uniform or dose-dependent effects across all GLP-1 receptor agonists. Important clinical variables such as gastric emptying times, standardized symptom scores, inflammatory biomarkers, and lung function parameters were incompletely captured, limiting mechanistic exploration.

Confounding by indication is a major limitation of this analysis. In routine clinical practice, clinicians may preferentially avoid GLP-1 receptor agonists in patients with more severe or refractory gastroparesis due to concerns about symptom exacerbation. As a result, patients receiving GLP-1 therapy may represent a population with milder disease, which could bias observed associations toward favorable outcomes despite propensity matching. Gastroparesis and outcome diagnoses were identified using ICD-10 and CPT codes rather than clinical adjudication. Accordingly, misclassification of outcomes, particularly between clinically overlapping entities such as pneumonitis and aspiration pneumonia, is possible and could not be systematically distinguished within the dataset. Objective gastric emptying studies, symptom severity scores, and phenotype validation were not available. Misclassification of both exposure and outcomes is therefore possible and may have influenced results. Detailed data on treatment duration, adherence, dose changes, glycemic indices, and episodes of hypoglycemia and persistence were not consistently available and continuous exposure throughout follow-up could not be fully verified, which limits interpretation of temporal and mechanistic associations. Data on gastroparesis severity markers, including hospitalization frequency, prokinetic medication use, and symptom burden, were not available and therefore could not be incorporated into adjusted or sensitivity analyses. Follow-up was limited to 6 months; infections occurring beyond this period were classified as new incident events rather than sequelae related to baseline gastroparesis. A formal negative control outcome analysis could not be performed because no outcome meeting criteria for clinical independence from both GLP-1 prescribing behavior and infection risk was reliably captured across contributing health systems. Limited granularity in exposure timing precluded assessment of time-varying exposure or dose–response relationships.

Finally, the generalizability of these findings may be limited beyond healthcare systems represented within the TriNetX network, which predominantly comprises North American institutions. Prescribing patterns for GLP-1 receptor agonists, diagnostic thresholds, and coding practices may differ in other regions or in health systems with alternative reimbursement structures or clinical guidelines. As a result, the observed associations may not fully extrapolate to non-North American populations or settings with substantially different patterns of GLP-1 use. Nonetheless, TriNetX includes a large and heterogeneous patient population across multiple institutions, and the consistency of associations observed across several infectious outcomes supports the internal validity and relevance of these findings within similar real-world clinical contexts.

### 4.6. Future Directions

Future research should prospectively evaluate the relationship between GLP-1 therapy and infectious outcomes in patients with diabetic gastroparesis using granular clinical data. Randomized or pragmatic trials incorporating symptom assessment, gastric emptying metrics, and inflammatory biomarkers could clarify mechanistic pathways. Experimental and translational work—integrating microbiome, metabolomic, and immunologic profiling—may further elucidate how incretin-based therapies modulate host defense in metabolic disease and critical illness [21,27,28,29,30,33]. Such studies could ultimately inform therapeutic stratification, identifying which subsets of gastroparesis patients derive the greatest benefit from GLP-1 therapy while minimizing gastrointestinal side effects.

### 4.7. Final Remarks

In summary, this real-world analysis demonstrates that GLP-1 receptor agonist therapy is associated with lower rates of pulmonary and systemic infectious complications in patients with diabetes and gastroparesis. Despite concerns regarding delayed gastric emptying, the overall clinical trajectory shows associations with pneumonitis, sepsis, and bacteremia suggesting broader anti-inflammatory and metabolic benefits [23,24,25,26,27]. These findings challenge prior assumptions about the risks of GLP-1 therapy in gastroparesis and support further investigation into its potential protective role in mitigating infection-related morbidity in complex diabetic populations [23,24,25,26,27,32,33].

## 5. Conclusions

GLP-1 receptor agonist therapy was significantly associated with pulmonary and systemic infectious complications among patients with diabetes and gastroparesis. These findings suggest that the overall benefits of GLP-1 therapy—spanning metabolic, vascular, and anti-inflammatory domains—may outweigh theoretical risks related to delayed gastric emptying. Further prospective and mechanistic studies are warranted to validate these associations and guide evidence-based management in this complex population.

## Figures and Tables

**Table 1:** Baseline demographic, diagnostic, and medication characteristics of Cohort 1 (diabetes with gastroparesis without GLP-1 therapy) and Cohort 2 (diabetes with gastroparesis with GLP-1 therapy) after propensity score matching.

Cohort 1 (N = 23,371) and Cohort 2 (N = 23,371) Characteristics After Propensity Score Matching						
Demographics						
Cohort		Mean ± SD	Patients	% of Cohort	*p*-Value	Std diff.
1	Current Age	61.2 ± 15.1	23,371	100%	<0.001	0.038
2		60.7 ± 12.7	23,371	100%		
1	Age at Index	57.0 ± 15.1	23,371	100%	<0.001	0.042
2		56.4 ± 12.5	23,371	100%		
1	Male		7166	30.70%	0.880	0.001
2			7151	30.60%		
1	Female		16,198	69.30%	0.960	<0.001
2			16,203	69.30%		
1	Black or African American		4775	20.40%	0.167	0.013
2			4896	20.90%		
1	White		15,270	65.30%	0.573	0.005
2			15,212	65.10%		
1	American Indian or Alaska Native		156	0.70%	0.351	0.009
2			140	0.60%		
1	Unknown Race		1188	5.10%	0.769	0.003
2			1202	5.10%		
1	Native Hawaiian or Other Pacific Islander		188	0.80%	0.368	0.008
2			171	0.70%		
1	Unknown Ethnicity		2324	9.90%	0.170	0.013
2			2236	9.60%		
1	Not Hispanic or Latino		18,136	77.60%	0.991	<0.001
2			18,135	77.60%		
1	Hispanic or Latino		2911	12.50%	0.216	0.011
2			3000	12.80%		
1	Other Race		1198	5.10%	0.834	0.002
2			1188	5.10%		
1	Asian		596	2.60%	0.312	0.009
2			562	2.40%		
**Diagnosis**						
1	Other chronic obstructive pulmonary disease		4053	17.30%	0.391	0.008
2			3983	17.00%		
1	Chronic kidney disease (CKD)		7397	31.70%	0.277	0.01
2			7288	31.20%		
1	Overweight and obesity		14,306	61.20%	0.001	0.031
2			13,952	59.70%		
**Medication**						
1	Insulin and analogues		17,295	74.00%	0.156	0.013
2			17,160	73.40%		
1	Biguanides		14,061	60.20%	0.029	0.02
2			13,830	59.20%		
1	Sulfonylureas		6483	27.70%	0.741	0.003
2			6515	27.90%		
1	Alpha glucosidase inhibitors		158	0.70%	0.578	0.005
2			168	0.70%		
1	Thiazolidinediones		1882	8.10%	0.588	0.005
2			1914	8.20%		
1	Dipeptidyl peptidase 4 (DPP-4) inhibitors		4139	17.70%	0.039	0.019
2			4311	18.40%		
1	Sodium–glucose co-transporter 2 (SGLT2) inhibitors		3819	16.30%	<0.001	0.065
2			4400	18.80%		

**Table 2:** Comparative analysis of pulmonary and infectious outcomes among patients with diabetes and gastroparesis receiving versus not receiving GLP-1 receptor agonist therapy.

Outcome	Incidence (No GLP-1)	Incidence (GLP-1)	Risk Ratio (95% CI)	Hazard Ratio (95% CI)	*p*-Value	Absolute Risk Difference (ARD)	NNT/NNH
Pneumonitis	3.60%	2.50%	1.43 (1.29–1.59)	1.76 (1.58–1.95)	<0.001	1.10%	91
Pneumonia	13.20%	12.20%	1.08 (1.03–1.13)	1.34 (1.27–1.41)	<0.001	1.00%	100
Mechanical Ventilation	4.40%	3.30%	1.33 (1.22–1.46)	1.63 (1.49–1.79)	<0.001	1.10%	91
Ventilator-Associated Pneumonia	0.18%	0.20%	0.94 (0.62–1.42)	1.14 (0.75–1.73)	0.750	−0.02%	5000 (NNH)
Sepsis	12.80%	11.10%	1.16 (1.10–1.22)	1.44 (1.37–1.52)	<0.001	1.70%	59
Bacteremia	5.20%	4.40%	1.19 (1.10–1.29)	1.46 (1.35–1.59)	<0.001	0.80%	125
Empyema	0.40%	0.30%	1.49 (1.10–2.03)	1.80 (1.32–2.45)	0.011	0.10%	1000
ARDS	0.80%	0.70%	1.16 (0.95–1.43)	1.41 (1.15–1.74)	0.001	0.10%	1000
Lung Abscess	0.20%	0.10%	1.12 (0.70–1.78)	1.36 (0.85–2.16)	0.640	0.10%	1000
PEG/Nasal Tube Feeding	0%	0%	—	—	—	0%	—

## Data Availability

The original contributions presented in this study are included in the article. Further inquiries can be directed to the corresponding author(s).

## Supplementary Materials

The following supporting information can be downloaded at: https://www.mdpi.com/article/10.3390/arm94020020/s1. Supplementary Table S1. Additional baseline comorbidities of the study cohorts after propensity score matching; Supplementary Table S2. Diagnostic and procedural codes used in the study.
