# Supplementary material for: Glucagon-like Peptide-1 Receptor Agonist Therapy and Risk of Pulmonary and Systemic Infections in Diabetic Gastroparesis: A Propensity-Matched Cohort Study
Source: Adv Respir Med. 2026 Mar 24;94(2):20. doi: 10.3390/arm94020020 (PMC13113843; doi:10.3390/arm94020020)
Supplement: Supplementary file 1 [file arm-94-00020-s001.zip › arm-4079553-supplementary.pdf]

Supplementary Materials

The following supplementary materials provide additional methodological details and supporting data for this study.

Supplementary Table S1. Additional baseline comorbidities of the study cohorts after propensity score matching.

Diagnosis	Cohort 1 (%)	Cohort 2 (%)	p-value	Std diff.
Acute myocardial infarction	10.6%	10.6%	0.916	0.001
Heart failure	22.7%	22.4%	0.419	0.007
Peripheral vascular disease	13.8%	13.5%	0.300	0.010
Cerebral infarction	9.3%	9.2%	0.701	0.004
Dementia	2.0%	2.0%	0.508	0.006
Connective tissue disease	3.8%	3.9%	0.866	0.002
Peptic ulcer disease	3.1%	3.2%	0.652	0.004
Liver disease	29.8%	30.1%	0.558	0.005
Hemiplegia	2.5%	2.6%	0.598	0.005
Neoplasms	43.5%	43.5%	0.963	<0.001
HIV	0.7%	0.8%	0.387	0.008

Supplementary Table S2. Diagnostic and procedural codes used in the study.

Outcome	Coding System	Codes
Pneumonia	ICD-10	J13–J18; 483
Pneumonitis	ICD-10	J69
VAP	ICD-10	J95.851
Mechanical ventilation	CPT	1015098
Sepsis	ICD-10	A40–A41
Bacteremia	ICD-10	R78.81
Empyema	ICD-10	J86
Lung abscess	ICD-10	J85
ARDS	ICD-10	J80
PEG	SNOMED	229912004
Enteral feeding	SNOMED	183028005
Diabetes	ICD-10	E08–E13
Gastroparesis	ICD-10	K31.84
GLP-1 RA exposure	ATC	A10BJ
